# Prognosis prediction models for post-stroke depression: a protocol for systematic review, meta-analysis, and critical appraisal

**DOI:** 10.1186/s13643-024-02544-x

**Published:** 2024-05-22

**Authors:** Lu Zhou, Lei Wang, Gao Liu, EnLi Cai

**Affiliations:** School of Nursing, Yunnan University of Chinese Medicine, Kunming, China

**Keywords:** Stroke, Depression, Models, Protocols, Guidelines

## Abstract

**Introduction:**

Post-stroke depression (PSD) is a prevalent complication that has been shown to have a negative impact on rehabilitation outcomes and quality of life and poses a significant risk for suicidal intention. However, models for discriminating and predicting PSD in stroke survivors for effective secondary prevention strategies are inadequate as the pathogenesis of PSD remains unknown. Prognostic prediction models that exhibit greater rule-in capacity have the potential to mitigate the issue of underdiagnosis and undertreatment of PSD. Thus, the planned study aims to systematically review and critically evaluate published studies on prognostic prediction models for PSD.

**Methods and analysis:**

A systematic literature search will be conducted in PubMed and Embase through Ovid. Two reviewers will complete study screening, data extraction, and quality assessment utilizing appropriate tools. Qualitative data on the characteristics of the included studies, methodological quality, and the appraisal of the clinical applicability of models will be summarized in the form of narrative comments and tables or figures. The predictive performance of the same model involving multiple studies will be synthesized with a random effects meta-analysis model or meta-regression, taking into account heterogeneity.

**Ethics and dissemination:**

Ethical approval is considered not applicable for this systematic review. Findings will be shared through dissemination at academic conferences and/or publication in peer-reviewed academic journals.

**Systematic review registration:**

PROSPERO CRD42023388548.

**Supplementary Information:**

The online version contains supplementary material available at 10.1186/s13643-024-02544-x.

## Introduction

Stroke is a serious public health concern worldwide, with elevated rates of mortality, disability, and recurrence [1]. Post-stroke depression (PSD) refers to any depressive state that occurs after a stroke, which is the most common neuropsychiatric disorder [2]. The prevalence of PSD ranges from 11 to 41%, with a cumulative incidence of 65%, of which roughly 14% are diagnosed with major depressive disorder (MDD) [1, 3, 4]. PSD is increasingly becoming a research hotspot due to its severe negative effects and economic burden [5].

While recovery from depression after a stroke within a year improves functional outcomes and quality of life [6, 7], PSD is linked to higher mortality, poorer recovery, more pronounced cognitive impairments, heavier financial burden, and lower quality of life than stroke without depression [8], indicating that depression hinders functional recovery after a stroke [9]. PSD can manifest at any point following a cerebrovascular event. It affects roughly one-third of stroke survivors and is notably associated with compromised functional recovery and heightened mortality rates. Thus, early screening and risk stratification interventions for stroke survivors at risk for depression are essential to adequately understand the mechanisms and development of symptomatology and even to change the prognosis. However, PSD arises from the complex interplay of neurobiological and psychosocial factors [1], exhibiting differential effects across various time frames post-stroke. The intricate interaction mechanisms and dynamic evolution of these factors throughout the development of PSD have posed enduring challenges within academic discourse. Consequently, this complexity contributes to suboptimal predictive dynamics and precision in PSD assessment.

Specifically, the diagnosis of PSD is primarily reliant on the Diagnostic and Statistical Manual of Mental Disorders (DSM) guidelines, in conjunction with a range of instruments measuring depression [10], but instruments have limitations in screening for PSD, such as insufficient clinical applicability or poor specificity [11]. In addition, PSD has been frequently under-diagnosed and under-treated due to the pathophysiological mechanisms of PSD not being fully understood [12], causing a sub-optimal prognosis for stroke survivors [13]. Nevertheless, the heightened administration of pharmacological treatment involving antidepressants, specifically escitalopram and fluoxetine, has demonstrated effectiveness in individuals who exhibit a high risk of PSD. But it is essential to note that such treatment may pose an excessive risk of harm in those who exhibit a lower risk of PSD. Notably, fluoxetine cannot improve depressive symptoms in PSD patients [13, 14], and these therapies lack risk stratification. Thus, identifying prediction variables (e.g., biomarkers or psychosocial factors, as well as demographic and clinical characteristics of patients) associated with an increased risk of PSD occurrence and then developing multivariable prediction models is one of the promising PSD prevention strategies [15].

Currently, the construction, validation, and updating of predictive models are gaining attention in clinical research [16]. Prediction models are formal combinations of multiple predictors that estimate the probability of an individual currently having a certain disease (diagnostic model) or having a certain outcome in the future (prognostic model) through a mathematical formula [17], from which risks for specific endpoints can be calculated for individual patients to facilitate the dissemination of preventive interventions, provide patient counseling, and establish clinical guidelines and policies [18, 19]. This study will focus on prognostic models.

Previous work has shown prediction models provide more accurate and less variable estimates of risk compared to more subjectively made predictions [20], but the methodology of model development is key to ensuring predictive performance. Although an increasing number of prognostic prediction models for PSD have been published [21, 22], there has been limited advancement in the development of prognostic models for the stratification of PSD and MDD in stroke survivors [9], which are mainly based on clinical characteristics and biological markers ignoring psychosocial data support [23] causing the limited clinical predictive value. In addition, most of the existing prediction models are opportunistic and have been rarely used or even mentioned in clinical guidelines [24]. Only a small proportion of these models have been evaluated for their performance in data from other participants. Further, research design flaws, insufficient statistical methods, and incomplete reporting hinder the clinical application of these models. According to the PROGRESS group, significant heterogeneity exists among studies, the inclusion and exclusion criteria are too narrow, stroke type (ischemic or hemorrhagic) is not reported, blinding is rarely reported, preset cutoff values are not reported, multiple predictive models are rarely compared in the same population, and the appraisal of models across different languages, races/ethnicities, and cultures is lacking [15]. These factors point to significant waste in research, including both financial and scientific resources [25].

As the research on PSD prognostic prediction models continues to grow annually, there are varying emphases on the content, format, performance, and modeling approaches. The abundance of available clinical research data poses challenges for clinicians in extracting evidence, making it difficult to discern the most targeted predictive prognostic models to assist clinical decision-making and determine best practices from independently published literature. Furthermore, after preliminary searches in the PROSPERO database, Cochrane systematic review database, and JBI evidence synthesis, no completed or ongoing systematic reviews or scoping reviews were identified.

Thus, a comprehensive review and overview of existing PSD models is necessary to clarify their predictive performance, advantages, disadvantages, usage characteristics, and methodology. This will provide evidence-based support for practitioners in selecting models, while also promoting the development, validation, and updating of prognosis prediction models for PSD.

### Research aims

The planned study aims to conduct a systematic review of all available evidence regarding the current prognostic models for PSD and to identify which prognostic prediction models have been developed, establishing the most effective and best performance model to predict PSD, while informing clinical decision-making. The specific aims of this systematic review are:

1. To ascertain the existing prognostic prediction models for PSD.
2. To qualitatively characterize the qualitative properties of the included prognostic prediction models.
3. To summarize and compare the current prognostic models and their predictive performance.
4. To critically appraise the studies identified for inclusion, particularly the research methodology and reporting methods.

## Methods and analysis

The present protocol was formulated in adherence to the Preferred Reporting Items for Systematic Review and Meta-Analysis Protocol (PRISMA-P) guidelines and was duly registered with PROSPERO, the international prospective register of systematic reviews (Supplementary Material 1 contains the PRISMA-P checklist for reference) [26, 27].

A systematic review of prognostic prediction modeling studies for PSD will be conducted and will be in accordance with the guidelines established by the Cochrane Prognosis Methods Group (PMG) and PROGnosis RESearch Strategy (PROGRESS) throughout all stages of the process [28–30]. Certain specific steps and models, for instance, framing, critical appraisal, and the assessment of the risk of bias, will be conducted by employing the CHARMS checklist (critical appraisal and data extraction for systematic reviews of prediction modeling studies) [31] and the PROBAST (Prediction model Risk Of Bias ASsessment Tool) with four domains (i.e., participants, predictors, outcome, and analysis) [32, 33]. Moreover, for predictive modeling studies applying machine learning techniques, study selection and evidence appraisal will be based on the metrics and statements highlighted and extended in the Transparent Reporting of a multivariable prediction model of Individual Prognosis Or Diagnosis—Artificial Intelligence (TRIPOD-AI) and PROBAST-AI being developed [34].

### Eligibility criteria

The outline of the review data and study selection were defined according to the CHARMS checklist (key items to guide the framing of the review aim, search strategy, and study inclusion and exclusion criteria) [31] detailed in Table 1.

**Table 1:** The CHARMS checklist

Item	Comments
1. Prognostic versus diagnostic prediction model	The aim is to predict future events (prognostic prediction model)
2. Intended scope of the review	Models to inform healthcare professionals’ clinical decision-making
3. Type of prediction modeling studies	All study types, i.e., prediction model development studies with and without external validation and external model validation studies with or without model updating
4. Target population to whom the prediction model applies	Survivors with stroke diagnosed according to each included study
5. Outcome to be predicted	• Assess the presence of post-stroke depression (morbidity) Mortality (primary) • PSD-related outcomes (secondary) PSD-related functional status PSD-related health status PSD-related quality of life
6. Time span of prediction	Depending on the duration of treatment and follow-up, the duration of prediction may differ across studies. However, it is anticipated that the majority of studies will assess endpoints within a range of 1 to 12 months
7. Intended moment of using the model	The proposed model is intended for utilization within clinical or hospital environments during the period of stroke diagnosis. Its purpose is to assist in the process of screening, providing targeted treatment, and offering programmatic support to stroke survivors who are at the highest risk of developing PSD

The inclusion criteria are as follows: (1) studies that develop or validate prognostic models (e.g., machine learning and Cox models), whether or not they include external validation; (2) study populations that involve research on ischemic or hemorrhagic stroke; (3) primary outcome measures indicating whether PSD occurred; (4) secondary outcome measures related to PSD, such as functional status, health status, quality of life, or mortality; (5) studies with a sample size of adequate power to detect small effects, with a goodness-of-fit statistic of over 0.99 for both closed and non-closed models; (6) cross-sectional and longitudinal primary research or literature research; (7) studies that report statistical models or instruments and their prediction indicators for predicting an individual’s risk of a future outcome (i.e., prognostic prediction model); (8) other names for prediction models include prognostic models, prognostic or prediction indices or rules, risk or clinical prediction models, and predictive models; and (9) prediction models are used to estimate the probability of a specific outcome occurring and can be reported using either absolute probability or relative risk score terms [35].

The exclusion criteria are as follows: (1) diagnostic prediction models; (2) evaluation of the predictive value of more than one variable, but without reporting subgroups or evaluation outcomes; (3) study population not related to stroke or studying a combined population with missing grouping results, or the study population only includes patients with individual or multiple complications of vascular damage (infarction, WMH, atrophy), such as cognitive impairment or dementia; (4) targeting depression occurring before stroke onset; and (5) presented as literature reviews, meta-reviews, protocols, theses, quality improvement activities, editorial comments, or letters, or not available in full text.

There will be no restrictions on year or language. In instances where multiple studies reported results from the identical cohort concerning a specific outcome measure, the data from the study that encompassed the largest patient population will be selected for analysis. Alternatively, if the studies involved an equal number of patients, the data from the earliest published study will be utilized.

### Information sources

A search will be conducted in the following electronic databases: Ovid MEDLINE® Epub Ahead of Print, In-Process, Other Non-Indexed Citations, Daily Update; Embase Classic—Ovid®; Coverage: 1946 to present. The reference list of the included studies will undergo a meticulous manual search to identify any additional potentially relevant citations and a manual search will be conducted with the Google Scholar web search engine.

### Search strategy

The search strategy will be devised for MEDLINE using the OvidSP platform, incorporating Medical Subject Headings (MeSH) and relevant keywords to enhance the efficacy of the search process (MeSH terms are available in Supplementary Material 2). Specifically, subject indexing terms will include a combination of the following five aspects of the PICOS system search construct [35, 36]: #1 Population search AND #2 Index search AND #3 Comparator search AND #4 Outcomes search NOT #5 Study design-exclusion filter.

All model development studies will be back-citation-searched to identify potentially relevant external validation studies. Subsequently, a comprehensive review of all retrieved studies will be performed to ascertain their suitability for inclusion in the analysis. References identified by the search strategy will be entered into Endnote bibliographic software to screen the selected articles.

### Study records

#### Data management

Upon exportation from electronic databases, all search results will be subsequently imported into Covidence, a systematic review management platform, to facilitate efficient and organized review and analysis [37], available at https://www.covidence.org, and duplicates will be removed.

#### Study selection

Based on the established eligibility criteria for article selection, one author (L.Z.) will test the retrieve strategy across all the databases while two authors (G.L. and L.W.) will independently screen the titles and abstracts. The search results will be then screened a second time, in duplicate. Potential disagreements regarding the inclusion of an article will be resolved through a discussion but, in case of differences, a third researcher (EL.C.) decides whether to include an article. If there is no sufficient data to determine eligibility, additional information will be obtained from the study authors; if missing data cannot be obtained, studies will be excluded from the analysis. But the report with the highest risk of bias will also be removed if data from the identical samples are related to the same model testing.

#### Data collection process

The data will be extracted independently across the included studies by two reviewers (L.Z. and G.L.) using a standardized electronic form developed with reference to the CHARMS checklist (relevant items to extract from individual studies in a systematic review of prediction models for purposes of description or assessment of risk of bias or applicability) that is available in Supplementary Material 3 [31]. Moreover, the data items in the checklist will adapt to the specific clinical question, for instance, aims; data source; participants; stakeholders; algorithms; predicted outcomes; potential predictors; sample size; missing data; model development; model performance, including properties of discrimination with confidence intervals, calibration, classification, and overall performance; final multivariable models; interpretation of presented models; and model evaluation. Through discussions between the co-investigator (EL.C.) and two reviewers (L.Z and L.W.), the two data collection sheets will be reconciled into one data set. Any disagreement or uncertainty will be resolved by discussion among reviewers to reach a consensus, if required, by consulting another author of the review team (EL.C.).

### Critical appraisal

PROBAST will be used to analyze the methodological quality and relevance of participants, predictors, and outcomes from each included study to the review topic in a systematic assessment [16]. With a total of 20 signaling questions, this instrument comprises four domains: participants, predictors, results, and analysis. Domains were scored as “high,” “low,” or “unclear” risk of bias. Two reviewers (L.Z. and G.L.) will independently apply the tool to rate the risk of bias and applicability of each included study of the 10 studies. The kappa coefficient for inter-rater reliability should be over 0.8 [38]. Any disagreement will be resolved by discussion. Graphical representations will be utilized to present the findings of each study.

### Data synthesis

#### Evidence synthesis

The initial methodology will involve utilizing a narrative synthesis approach to systematically detail the characteristics and quantitative data obtained from the studies that have been included. Specifically, the qualitative/heterogeneous outcomes of studies, including predictors, performance measures, classification measures, measures of uncertainty, and a descriptive analysis of key items [30], will be summarized qualitatively. Results will be presented in tabular form with each study to facilitate comparison.

#### Meta-analysis

The homogeneous outcomes of the same prediction model which meet the following criteria will be statistically analyzed in meta-analysis: (1) across ≥ 2 studies; (2) the identical category of prediction modeling study, specifically either development or validation; and (3) the follow-up periods for the primary outcome(s) are considered similar. While conducting the meta-analysis, it is possible to combine re-scaled measures of model performance which have similar outcomes. It will be typically accomplished via a random-effects meta-analysis approach, using restricted maximum likelihood estimation. Additionally, the Hartung-Knapp-Siddik-Jonkman method will be used to derive confidence intervals. Where feasible, 95% prediction intervals will be estimated. The performance of the prognostic prediction model will be based on the following measures [30, 39], detailed in Table 2. Additionally, where possible, we will employ multivariate meta-analysis for jointly synthesizing calibration and discrimination performance, while accounting for their correlation.

**Table 2:** Summary of measuring performance of prognostic prediction models

Items	Performance measures/statistics	Values for better performance	Visualization
Discrimination	AUC-ROC	Higher	ROC curve
	ACC	Higher	
	BER	Lower	
	*D* statistics	Lower	
	MCC	Higher	
	F1 score	Higher	
	Log-rank	Lower, *p* > *.05*	
	AUC-PRC	Higher	PRC curve
Calibration	Calibration curve, slope, and intercept	Slope closer to 1 and intercept closer to 0	Calibration plot
	Reliability-deviation	Lower	
	Reliability–within-bin variation	Lower	
	Reliability–within-bin covariance	Higher	
	Resolution	Higher	
	Predictive range	Higher	
	Emax	Lower	
	Eavg	Lower	
	Hosmer–Lemeshow test	Higher	
	Total O:E ratios	Higher	
Classification	RMSE via neighborhood estimate	Lower	Reclassification scatter plot
	*R*^2^ statistic	Higher	
	TPR	Higher	
	TNR	Higher	
	NRI	Higher	
	IDI	Higher	
Overall performance	Brier score	Lower	
	Brier skill score	Higher	
	Prediction squared error	Lower	
	Decision Curve Analysis	/	DCA plot

*AUC-ROC* Area under receiver operating characteristic curve, *TPR* Sensitivity, *ACC* Accuracy, *BER* Balanced error rate, *MCC* Matthews correlation coefficient, *TNR* Specificity, *AUC-PRC* Area under precision-recall curve, *RMSE* Root-mean-squared error, *NRI* Net reclassification improvement, *IDI* Integrated discrimination improvement

#### Sensitivity analysis and investigation of heterogeneity

To ascertain the robustness of the findings, sensitivity analyses will be conducted, wherein studies deemed to have a significant or uncertain risk of bias will be excluded. The *I*^2^ statistic for univariate meta-analysis models and sub-group analyses will be employed to explore heterogeneity between studies. Between-effects heterogeneity will be estimated via restricted maximum-likelihood *I*^2^ and tau^2^ statistics. Potential sources of considerable between-effects heterogeneity will be investigated by conducting a meta-regression analysis (*p* < 0.05). If possible, the sub-group analysis will be based on:

1. Stroke types—ischemic or hemorrhagic.
2. Risk factors—biomarkers or psychosocial factors.
3. Depression types—PSD or MDD.
4. Modeling techniques—machine learning or non-machine learning.
5. Follow-up duration.
6. Region—based on the Organisation for Economic Co-operation and Development classification, that is, low/middle-income and high-income countries.

The meta-analysis process will be conducted in the metareg module in Stata 13.0 regarding the Meta-analysis of Observational Studies in Epidemiology (MOOSE) group guidelines [40].

### Reporting findings

The findings of this systematic review will be reported in adherence to the TRIPOD (Transparent Reporting of a multivariable prediction model for Individual Prognosis Or Diagnosis) guideline [41, 42] and the PRISMA statement (Preferred Reporting Items for Systematic Reviews and Meta-Analyses) [43].

## Discussion

The planned study will be the first systematic review to evaluate existing evidence regarding prognostic prediction models (including machine learning algorithms, statistical models, and clinical risk scales) aimed at post-stroke depression for secondary prevention. The occurrence mechanism of PSD is complex and diverse. Currently, there is a lack of a gold standard for diagnosing PSD, and screening instruments have certain limitations, resulting in a relatively high rate of missed diagnoses. Although numerous PSD prediction models have been developed at this stage, most of the prediction models are not developed, validated, and assessed based on guidelines for predictive research [34]. This has led to significant biases in risk estimation and serious deficiencies in statistical methods, as well as a lack of internal and external validation [11], affecting the performance and applicability of the models and resulting in less-than-ideal accuracy and precision in clinical PSD prediction. Additionally, at present, there is a lack of systematic reviews and evaluations of PSD prediction models, which hinders relevant practitioners in selecting, promoting, and applying these models. This systematic review refers to details of the foundation and evidence for further studies, which aimed at developing, verifying, implementing, and assessing prognostic prediction models for PSD within the four domains of the PROGRESS prognosis research framework [44]. Regarding the TRIPOD-AI and PROBAST-AI tool, incorporating insights from these forthcoming extensions could enhance the review’s comprehensiveness and relevance, especially concerning machine learning-based prognostic models, ultimately contributing to more robust and applicable prognostic models for PSD in secondary prevention.

The findings will facilitate the early identification of people at high risk for PSD, the identification of the most effective current prognostic prediction models based on the shown predictive accuracy, and the stratification of PSD severity to estimate the risk of MDD after stroke. This will be a significant step towards informing the clinical management of patients with an established stroke diagnosis. It is essential for accurate identification of PSD, translation of clinical research of high-quality evidence, and savings in healthcare resources. Additionally, it will promote the consideration of the broad continuum of risk related to this condition in routine clinical practice. At a health service level, prediction models with good performance and high clinical applicability would support a personalized risk-stratified model of care, which would ultimately better direct finite health resources to stroke survivors at high risk of PSD and most likely to benefit from intervention.

### Supplementary Information

**Supplementary Material 1. **

**Supplementary Material 2. **

**Supplementary Material 3. **

## Data Availability

This content has been supplied by the authors.
