# Supplementary material for: Prognosis prediction models for post-stroke depression: a protocol for systematic review, meta-analysis, and critical appraisal
Source: Syst Rev. 2024 May 22;13:138. doi: 10.1186/s13643-024-02544-x (PMC11110183; doi:10.1186/s13643-024-02544-x)
Supplement: Supplementary file 2 — Supplementary Material 2. [file 13643_2024_2544_MOESM2_ESM.pdf]

Appendix 2: An example of MeSH terms

("Cerebral Hemorrhage"[Mesh]) OR ("Stroke"[Mesh] OR "Stroke, Lacunar"[Mesh] OR "National Institute of Neurological Disorders and Stroke (U.S.)"[Mesh] OR "Hemorrhagic Stroke"[Mesh] OR "Embolic Stroke"[Mesh] OR "Thrombotic Stroke"[Mesh] OR "Ischemic Stroke"[Mesh] OR "Infarction, Posterior Cerebral Artery"[Mesh] OR "Brain Stem Infarctions"[Mesh] OR "Infarction, Middle Cerebral Artery"[Mesh] OR "Infarction, Anterior Cerebral Artery"[Mesh])

("Depression"[Mesh] OR "Depressive Disorder"[Mesh] OR "Depressive Disorder, Major"[Mesh] OR "Adjustment Disorders"[Mesh] OR "Bipolar Disorder"[Mesh] OR "Depressive Disorder, Treatment-Resistant"[Mesh] OR "Affective Disorders, Psychotic"[Mesh]) OR "Mood Disorders"[Mesh]
