# Supplementary material for: Prognosis prediction models for post-stroke depression: a protocol for systematic review, meta-analysis, and critical appraisal
Source: Syst Rev. 2024 May 22;13:138. doi: 10.1186/s13643-024-02544-x (PMC11110183; doi:10.1186/s13643-024-02544-x)
Supplement: Supplementary file 3 — Supplementary Material 3. [file 13643_2024_2544_MOESM3_ESM.pdf]

Appendix 3: CHARMS 2014 Relevant items to extract from individual studies in a systematic review of prediction models

Domain	Key items	Reported
SOURCE OF DATA	Source of data (e.g., cohort, case-control, randomized trial participants, or registry data)	
PARTICIPANTS	Participant eligibility and recruitment method (e.g., consecutive participants, location, number of centers, setting, inclusion and exclusion criteria)	
	Participant description	
	Details of treatments received, if relevant	
	Study dates	
OUTCOME(S) TO BE PREDICTED	Definition and method for measurement of outcome	
	Was the same outcome definition (and method for measurement) used in all patients?	
	Type of outcome (e.g., single or combined endpoints)	
	Was the outcome assessed without knowledge of the candidate predictors (i.e., blinded)?	
	Were candidate predictors part of the outcome (e.g., in panel or consensus diagnosis)?	
	Time of outcome occurrence or summary of duration of follow-up	
CANDIDATE PREDICTORS (OR INDEX TESTS)	Number and type of predictors (e.g., demographics, patient history, physical examination, additional testing, disease characteristics)	
	Definition and method for measurement of candidate predictors	
	Timing of predictor measurement (e.g., at patient presentation, at diagnosis, at treatment initiation)	
	Were predictors assessed blinded for outcome, and for each other (if relevant)?	
	Handling of predictors in the modelling (e.g., continuous, linear, non-linear transformations or categorised)	
SAMPLE SIZE	Number of participants and number of outcomes/events	
	Number of outcomes/events in relation to the number of candidate predictors (Events Per Variable)	
MISSING DATA	Number of participants with any missing value (include predictors and outcomes)	
	Number of participants with missing data for each predictor	
	Handling of missing data (e.g., complete-case analysis, imputation, or other methods)	
MODEL DEVELOPMENT	Modelling method (e.g., logistic, survival, neural network, or machine learning techniques)	
	Modelling assumptions satisfied	
	Method for selection of predictors **for inclusion** in multivariable modelling (e.g., all candidate predictors, pre-selection based on unadjusted association with the outcome)	
	Method for selection of predictors **during multivariable modelling** (e.g., full model approach, backward or forward selection) and criteria used (e.g., p-value, Akaike Information Criterion)	
	Shrinkage of predictor weights or regression coefficients (e.g., no shrinkage, uniform shrinkage, penalized estimation)	
MODEL PERFORMANCE	Calibration (calibration plot, calibration slope, Hosmer-Lemeshow test) and Discrimination (C-statistic, D-statistic, log-rank) measures with confidence intervals	
	Classification measures (e.g., sensitivity, specificity, predictive values, net reclassification improvement) and whether a-priori cut points were used	
MODEL EVALUATION	Method used for testing model performance: development dataset only (random split of data, resampling methods e.g. bootstrap or cross-validation, none) or separate external validation (e.g. temporal, geographical, different setting, different investigators)	
	In case of poor validation, whether model was adjusted or updated (e.g., intercept recalibrated, predictor effects adjusted, or new predictors added)	
RESULTS	Final and other multivariable models (e.g., basic, extended, simplified) presented, including predictor weights or regression coefficients, intercept, baseline survival, model performance measures (with standard errors or confidence intervals)	
	Any alternative presentation of the final prediction models, e.g., sum score, nomogram, score chart, predictions for specific risk subgroups with performance	
	Comparison of the distribution of predictors (including missing data) for development and validation datasets	
INTERPRETATION AND DISCUSSION	Interpretation of presented models (confirmatory, i.e., model useful for practice versus exploratory, i.e., more research needed)	
	Comparison with other studies, discussion of generalizability, strengths and limitations.	
